# Epstein Barr Virus and *Helicobacter pylori* Co-Infection Are Positively Associated with Severe Gastritis in Pediatric Patients

**DOI:** 10.1371/journal.pone.0062850

**Published:** 2013-04-24

**Authors:** María G. Cárdenas-Mondragón, Ricardo Carreón-Talavera, Margarita Camorlinga-Ponce, Alejandro Gomez-Delgado, Javier Torres, Ezequiel M. Fuentes-Pananá

**Affiliations:** Unidad de Investigación Médica en Enfermedades Infecciosas y Parasitarias (UIMEIP), Hospital de Pediatría, CMN Siglo-XXI, Instituto Mexicano del Seguro Social (IMSS), Mexico City, Mexico; Veterans Affairs Medical Center (111D), United States of America

## Abstract

**Background:**

*H. pylori* infection is acquired during childhood and causes a chronic inflammatory response in the gastric mucosa, which is considered the main risk factor to acquire gastric cancer (GC) later in life. More recently, infection by Epstein-Barr virus (EBV) have also been associated with GC. The role of EBV in early inflammatory responses and its relationship with *H. pylori* infection remains poorly studied. Here, we assessed whether EBV infection in children correlated with the stage of gastritis and whether co-infection with *H. pylori* affected the severity of inflammation.

**Methodology/Principal Findings:**

333 pediatric patients with chronic abdominal pain were studied. From them, gastric biopsies were taken and inflammation graded according to the Sydney system; peripheral blood was drawn and antibodies against EBV (IgG and IgM anti-VCA) and *H. pylori* (IgG anti-whole bacteria and anti-CagA) were measured in sera. We found that children infected only by EBV presented mild mononuclear (MN) and none polymorphonuclear (PMN) cell infiltration, while those infected by *H. pylori* presented moderate MN and mild PMN. In contrast, patients co-infected with both pathogens were significantly associated with severe gastritis. Importantly, co-infection of *H. pylori* CagA+/EBV+ had a stronger association with severe MN (PR 3.0) and PMN (PR 7.2) cells than cases with single *H. pylori* CagA+ infection.

**Conclusions/Significance:**

Co-infection with EBV and *H. pylori* in pediatric patients is associated with severe gastritis. Even single infections with *H. pylori* CagA+ strains are associated with mild to moderate infiltration arguing for a cooperative effect of *H. pylori* and EBV in the gastric mucosa and revealing a critical role for EBV previously un-appreciated. This study points out the need to study both pathogens to understand the mechanism behind severe damage of the gastric mucosa, which could identified children with increased risk to present more serious lesions later in life.

## Introduction

Persistent infections often lead to chronic inflammation, a well documented cancer risk factor. Gastric cancer (GC) generally starts with an inflammatory process mainly associated with infection by *Helicobacter pylori* (*H. pylori*) [1]. GC is the fourth most common type of cancer and the second cause of death by cancer world-wide, affecting particularly Asian and Latin American countries [2]. More recently, GC has also been associated with Epstein-Barr virus (EBV) but the role of the viral infection in early inflammatory gastric responses remains poorly studied.

*H. pylori* infects over 50% of the world population, with a higher prevalence in developing countries. Infection is usually acquired early in life; in Mexico, about 50% of children are infected by the age of 10 [3]. Inflammation after infection in children is usually associated with a low level of polymorphonuclear (PMN) and mononuclear (MN) cells infiltrating the gastric mucosa [4]. It has been suggested than the earlier the infection, the greater the risk to present GC later in life, conceivable because of a long lasting (decades) chronic inflammatory reaction to the infection [5].

Only a fraction of *H. pylori* infected individuals develop gastroduodenal disease: <15% peptic ulcer, <3% GC and <1% MALT lymphoma [6]. The outcome of *H. pylori* infection depends also on environmental, host and bacterial factors. Among the most important bacterial virulence factors is the pathogenicity island (CagPAI), which encodes a type IV secretion system (T4SS) that translocates the effector protein CagA into epithelial cells [7]. CagA activates multiple signaling pathways triggering cellular phenotypes associated with oncogenic transformation [7]. Moreover, transgenic mice expressing CagA develop adenocarcinomas of the digestive tract. Based on these data, CagA has been recognized as the first known bacterial oncoprotein [8], [9].

EBV infection has been consistently associated with several types of lymphoma, nasopharyngeal carcinoma (NPC) [10], [11] and more recently to GC [12], [13], [14]. EBV infection also occurs early in childhood and usually persists in B cells, with most infected individuals carrying the virus asymptomatically in a latent stage in these cells. It is not clear when EBV infects the gastric mucosa and whether infection induces an inflammatory reaction, as observed with *H. pylori*. EBV reactivation from infected B cells has been proposed to facilitate infection of the epithelial basolateral face [15]. In that scenario, the titer of anti-EBV antibodies against structural proteins has been proposed to correlate with the level of viral reactivation and as a prognostic marker in NPC [10], [16], [17].

To our knowledge, no studies have previously addressed whether EBV infection in children is associated with inflammation in the gastric mucosa or whether there exists a cooperative effect between EBV and *H. pylori* correlating with the severity of the inflammatory reaction. In this study, we analyzed antibodies against EBV and *H. pylori* in sera of pediatric patients with chronic abdominal pain. Our results strongly suggest that single infection by either EBV or *H. pylori* is associated with a mild to moderate inflammatory response in the gastric mucosa; however, co-infection with both pathogens is significantly associated with severe gastritis. Even infection with *H. pylori* cagA+ strains is not associated with severe inflammatory responses in the absence of EBV. These data argue for a previously unknown critical role of EBV infection in the induction of an inflammatory response in the gastric mucosa of children.

## Materials and Methods

### Ethics Statement

The IMSS National Research Ethics Committee approved this project. Parents or guardians of the patients were informed on the nature of the study and those willing to participate signed a written informed consent prior to specimen collection.

### Overview

#### Study population

The study included 333 pediatric patients (0–17****years old) attended because of recurring abdominal pain at the Gastroenterology unit, Pediatric Hospital of the Centro Medico Nacional SXXI, Instituto Mexicano del Seguro Social (IMSS), in Mexico City, between September 1994 and October 2001. Children were subjected to endoscopy and gastric biopsies were taken from antrum and corpus for histopathological diagnosis. Peripheral blood was also drawn and sera were stored at −80°C until tested for antibodies.

#### Data collected

Socio-demographic data and clinical information was registered in questionnaires at time of inclusion. The information collected included age, gender, clinical symptoms and clinical diagnosis based on endoscopy, histology and clinical presentation. Patients with antibiotic, proton pump inhibitor or antiacid treatments three weeks previous to sample collection were excluded from the study.

### Laboratory Methods

#### Histopathological examination

Two biopsy specimens were obtained from de antrum and two from the gastric corpus. One biopsy was used for *H. pylori* culture and the second was used for histologic examination. One biopsy from the antrum and one from the corpus were fixed in formaline, paraffin-embedded and stained with hematoxylin and eosin (HE). The inflammatory response was graded according to the Sydney system [18] by a single experienced pathologist. The parameters evaluated were *H. pylori* positivity, polymorphonuclear and mononuclear cell infiltration, which were graded comparing with published diagrams (analogue scales). Infiltration by polymorphonuclear and mononuclear cells was graded from absent to severe in both corpus and antrum, and the site with the higher infiltration was considered as the end result.

#### Collection of blood

A sample of venous blood (4****ml) was drawn from all patients. Stored serum samples were used to analyze IgG and IgM antibodies against EBV viral capsid protein (VCA), as well as IgG antibodies against *H. pylori* whole-cell extracts and against CagA protein by enzyme-linked immunosorbent assay (ELISA).

#### Determination of Anti-EBV VCA antibodies

Anti-EBV VCA antibodies were determined using ELISA commercial kits (HUMAN; Wiesbaden, Germany), for IgG anti-VCA (catalog 51204) and for IgM anti-VCA (catalog 51104) following manufacturer instructions. Briefly, 100 μl of the appropriate patient-serum dilution (1:100 for IgM and 1:20 for IgG) were deposited on the corresponding well with the VCA antigen already attached, and incubated for 1****hour (IgG) or 30****min (IgM) at 25°C. Next, the wells were washed four times with washing buffer, and 100 μl of peroxidase-conjugated anti-human IgG or IgM rabbit antibody were added and incubated for 30****min at 25°C. Plates were then washed five times and 100 μl of substrate reagent (3,3′,5,5′ tetramethylbenzidin (TMB) – hydrogen peroxide) were added and incubated for 15****min a 25°C in the dark. The reaction was then stopped with 100 µl of stop solution (sulphuric acid 0.5****mol/L) and the plates were read in an ELISA reader (Thermoscientific, multiskan ascent) to an absorbance of 450****nm. The reported value is the average of two independent assays. A subgroup of samples was done in quadruplicate using different lots of the ELISA kit to check for reproducibility. Calculations for antibody titers were done according to the manufacturer's instructions and the values are reported as HU units/ml. A patient was considered EBV seropositive when sera tested positive for either IgG and/or IgM antibodies.

#### Determination of antibodies anti-whole *H. pylori* extracts and anti-CagA

IgG antibodies against *H. pylori* and CagA were determined using ELISA tests previously validated in a Mexican population [19]. Wells were coated either with a sonicated extract of a mixture of three *H. pylori* strains isolated from Mexican patients (0.5 µg/well in 100 µl of carbonate buffer pH 9.6) or with recombinant CagA (0.1 µg/well in same buffer as above) and incubated at 4° overnight. Next, the plates were blocked with 200 µl of PBS-gelatin (0.1%) for *H. pylori* whole-extract and with PBS-milk (2.5%) for CagA and incubated at 4° overnight. The sera were diluted 1:1000 for *H. pylori* and 1:200 for CagA and 100 µl were added per well and incubated for one hour at 37°C. One hundred µl of a 1:1000 dilution of the anti-human IgG antibody-conjugated to alkaline phosphatase were added and plates were incubated for 30****min at room temperature, followed by 100 µl of p-nitrophenylphosphate substrate (Sigma; St. Louis MO USA. No. 2770) in glycine-MgCl2-ZnCl buffer. Plates were read in an ELISA reader at an absorbance of 405****nm. All samples were analyzed in duplicate and each plate included four positive and four negative sera samples. Patients were considered positive for *H. pylori* antibodies when ELISA units were ≥1.0, and for CagA when ELISA units were ≥1.5, according to the validated cut-offs [19].

### Statistical Analysis

The statistical differences of continuous variables between patient's groups were determined with the Student's *t* test or with the Mann-Whitney U test for non-normally distributed variables. Differences among three or more continuous variables were compared by one-way ANOVA, followed by Bonferroni test, or by the Kruskal-Wallis one-way test, followed by the Mann-Whitney U test. The differences between categorical variables were estimated using the Chi square (*X^2^*) test with Yate's continuity correction or the Fisher's exact test for small samples. Variables with more than two categories were analyzed using Mantel-Haenszel *X^2^* with linear tendency. Statistical significance was set at p≤0.05. The strength of associations between the inflammation grade's groups and IgG anti-VCA were estimated using prevalence ratios (PRs) and 95% confidence intervals (CIs).

## Results

### Characteristics of the patients studied

333 gastric samples from pediatric patients with chronic abdominal pain were studied, 197 (59.2%) were female and 136 (40.8%) male with an average and median age of 10.1±3.7 (mean ± SD) and 10****years, respectively (***Table*** **** ***1***). All patients were diagnosed with non atrophic gastritis (NAG) through histological examination; the morphology of the epithelial cells was not atypical and no glandular atrophy was found. The frequency of infection by EBV was 64.3% and by *H. pylori* 53.4%, whereas 33.9% presented antibodies against CagA. In most children the gastric mucosa presented with a mild infiltration of MN (78%) and no infiltration of PMN (75%); only 7.5% presented a severe infiltration of MN and 4.5% of PMN cells (***Table*** **** ***1***). Because of the low number of samples with severe activity, samples with a diagnostic of moderate and severe gastritis were combined for the rest of the analysis. The seroprevalence to *H. pylori* and EBV in the studied children according to age is described in ***Table*** **** ***S1***.

**Table 1:** Characteristics of the333 children studied.

Variable	Value
Age (mean±SD) Median	10.1±3.8 10
Sex, male/female (ratio)	136/197 (0.69)
EBV positive, n (%)	214 (64.3)
*H. pylori* positive, n (%)	178 (53.4)
*H. pylori* CagA+	112 (33.6)
Gastric mucosa cell infiltration	
MN infiltration (Inflammation):	
Mild, n (%)	260 (78)
Moderate, n (%)	48 (14.4)
Severe, n (%)	25 (7.5)
PMN infiltration (Activity):	
None, n (%)	250 (75.1)
Mild, n (%)	52 (15.6)
Moderate, n (%)	16 (4.8)
Severe, n (%)	15 (4.5)

### Degree of inflammation and infection with either EBV or *H. pylori*

We identified 214 children with EBV infection [179 were IgG and 35 were IgM positive (7 only IgM and 28 IgM and IgG)], 9.8% of these cases showed a severe MN infiltration, and 13.1% a moderate-severe PMN infiltration (***Table*** **** ***2***). All of the 35 children (28+7) positive for IgM were considered in primary infection and presented mild inflammation and none activity. On the other hand, 178 children were infected with *H. pylori* and of those 14% showed severe MN infiltration and 16.9% moderate-severe PMN infiltration. Among the 112 children infected with *H. pylori cagA*+ strains 18.8% had severe MN infiltration and 24.1% moderate-severe PMN infiltration. Whereas, none of the 66 children without infection presented severe MN nor PMN infiltration.

**Table 2:** Degree of infiltration of MN and PMN cells in the gastric mucosa of children according to infection with EBV and *H. pylori*.

Groups	Total	MN infiltration, n (%)			PMN infiltration, n (%)		
		Mild	Moderate	Severe	None	Mild	Moderate-severe
EBV positive	214	162 (75.7)	31 (14.5)	21 (9.8)	157 (73.4)	29 (13.6)	28 (13.1)
*H. pylori* positive	178	110 (61.8)	43 (24.1)	25 (14.0)	105 (58.9)	43 (24.1)	30 (16.9)
*H. pylori* CagA positiveEBV & *H. pylori* neg	112 66	54 (48.2) 63 (95.5)	37 (33) 3 (4.5)	21 (18.8) 0	55 (49.1) 61 (92.4)	30 (26.8)5 (7.6)	27 (24.1) 0

Significantly higher titers of anti-EBV antibodies were found only in mild PMN infiltration, suggesting that higher anti-EBV antibody titers are not indicative of more severe inflammation in pediatric gastritis (***Table*** **** ***S2***). On the other hand, a positive correlation of *H. pylori* antibody titers and the gastritis severity was observed as it has been previously reported [20], [21].

### Degree of inflammation related to the interaction between infection with EBV and *H. pylori*

**Figure 1:**
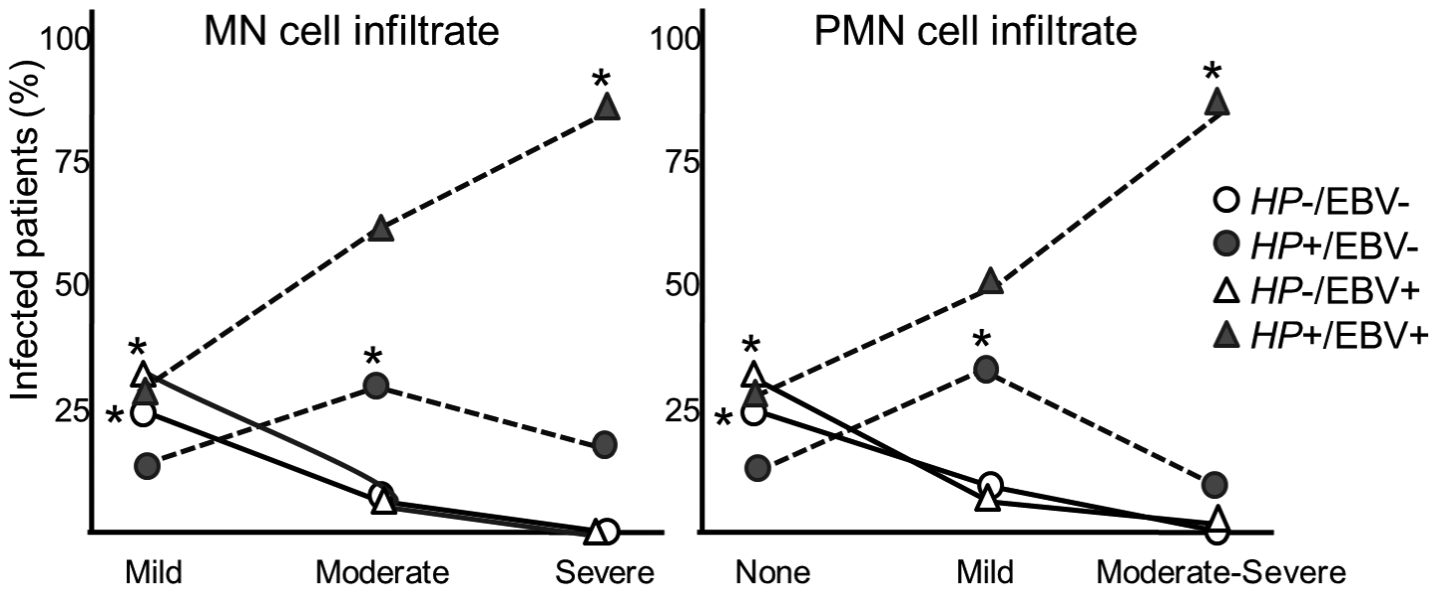
*H. pylory* and EBV infection frequencies in pediatric patients with gastritis according to the different grades of mononuclear (inflammation) and polymorphonuclear (activity) cell infiltration. Asterisks denote associations with statistical significance: *HP*−/EBV− and *HP*−/EBV+ groups with mild MN and no PMN (open circle and triangle), *HP*+/EBV− (grey circle) with moderate MN and mild PMN, and *HP*+/EBV+ (grey triangle) with severe MN and moderate-severe PMN.

We next addressed whether there was any interaction between infection with both *H. pylori* and EBV, and this analysis is presented in ***Table*** **** ***3*** (see also **Figure 1**). We found that most of the children with no infection (*HP*−/EBV−) and children with only EBV infection (*HP*−/EBV+) had a mild MN infiltration and no PMN in the gastric mucosa. Cases with moderate MN and mild PMN cells were more frequent in patients with only *H. pylori* infection (*HP*+/EBV−) and with both *H. pylori* and EBV infection (*HP*+/EBV+). In contrast, over 80% of the cases with severe MN and moderate-severe PMN infiltration occurred in patients with co-infection (*HP*+/EBV+).

**Table 3:** Degree of infiltration of mononuclear (MN) and polymorphonuclear (PMN) cells in the gastric mucosa of children according to co-infection with EBV and *H. pylori* (*HP*).

		MN infiltration, n (%)			PMN infiltration, n (%)		
Groups	Total	Mild	Moderate	Severe	None	Mild	Moderate-severe
*H. pylori* and EBV groups	333	260	48	25	250	52	31
*HP*−/EBV−	66 (19.8)	63 (24.2)	3 (6.3)	0	61 (24.4)	5 (9.6)	0
*HP*+/EBV−	53 (15.9)	35 (13.5)	14 (29.2)	4 (16)	32 (12.8)	18 (34.6)	3 (9.7)
*HP*−/EBV+	89 (26.7)	87 (33.5)	2 (4.2)	0	84 (33.6)	4 (7.7)	1 (3.2)
*HP*+/EBV+	125 (37.5)	75 (28.8)	29 (60.4)	21 (84)	73 (29.2)	25 (48.1)	27 (87.1)
CagA and EBV groups	178	110	43	25	105	43	30
*HP* CagA−/EBV−	19 (10.7)	16 (14.5)	2 (4.7)	1 (4)	13 (34.2)	5 (11.6)	1 (3.3)
*HP* CagA+/EBV−	34 (19.1)	19 (17.3)	12 (27.9)	3 (12)	19 (18.1)	13 (30.2)	2 (6.7)
*HP* CagA−/EBV+	47 (26.4)	40 (36.4)	4 (9.3)	3 (12)	37 (35.2)	8 (18.6)	2 (6.7)
*HP* CagA+/EBV+	78 (43.8)	35 (31.8)	25 (58.1)	18 (72)	36 (34.2)	17 (39.5)	25 (83.3)

**Figure 2:**
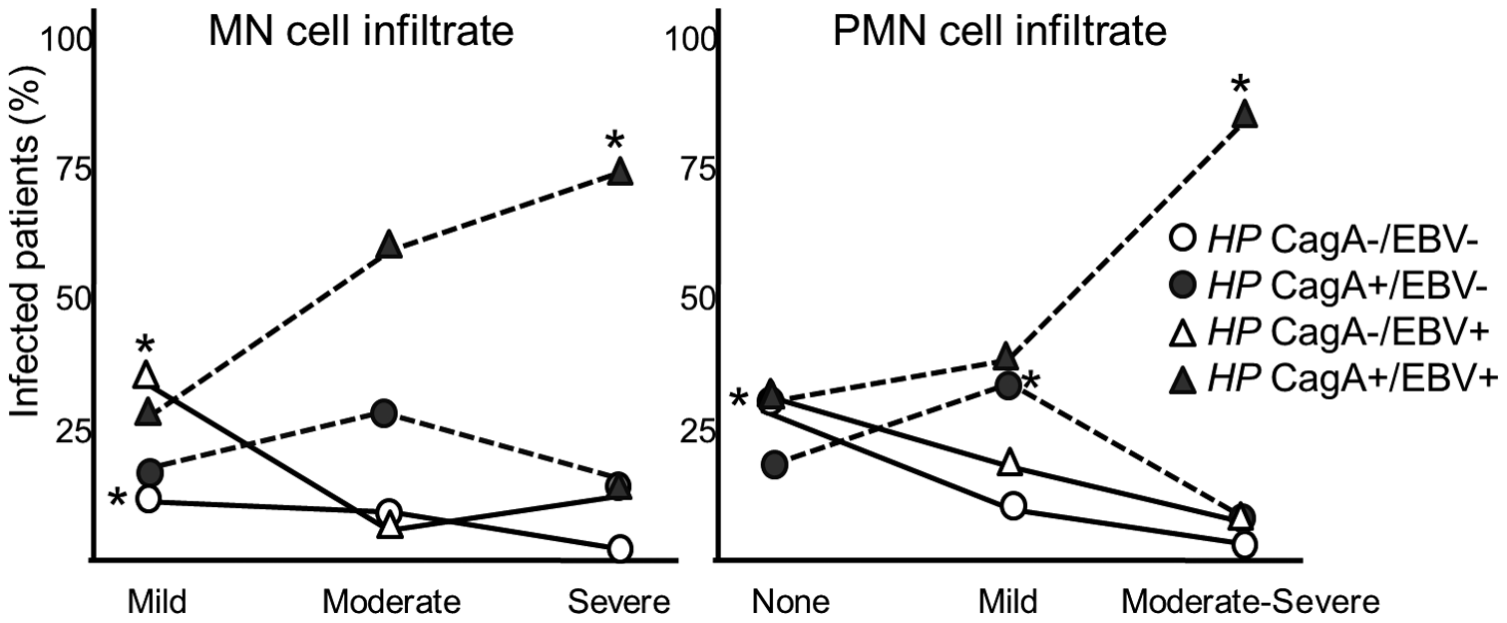
Infection frequencies and gastritis severity related to the presence of CagA. Asterisks denote associations with statistical significance: *HP* CagA−/EBV− and *HP* CagA−/EBV+ groups with mild MN and no PMN (open circle and triangle), *HP* CagA+/EBV− (grey circle) with mild PMN, and *HP* CagA+/EBV+ (grey triangle) with severe MN and moderate-severe PMN.

Positive interactions between *H. pylori* CagA and EBV according to the degree of inflammation were also analyzed and a similar result was observed (***Table*** **** ***3***), only *H. pylori* CagA+/EBV+ double positive patients were significantly associated with severe inflammation for both MN and PMN infiltrate (**Figure 2**). These results argue that *H. pylori* infection alone is not sufficient to develop severe gastritis.

To further confirm the combined effect of mixed EBV and *H. pylori* infection, we determined the significance of the association of co-infection with the cases showing a severe MN or moderate-severe PMN infiltration (***Table*** **** ***4***). In this analysis, to assess whether the observed association of EBV and *H. pylori* infection with severe gastritis could be influenced by age, patients were divided into two groups of age according to the median: ≤10 (group 1) and >10 (group 2), and prevalence ratios (PR) were adjusted by these age groups. When patients with co-infection (*HP*+/EBV+) were compared against patients with no infection (*HP*−/EBV−) PR values for severe MN and PMN infiltration were undefined because none of the uninfected children (*HP*−/EBV−) had severe MN or PMN infiltration. When patients with co-infection (*HP*+/EBV+) were compared against patients with single *H. pylori* infection (*HP*+/EBV−) PR's for severe infiltration were 2.2 for MN and 4.1 for PMN; this latter with statistical significance (p = 0.01).

**Table 4:** Age adjusted PR values for severe MN and moderate-severe PMN infiltration when compared between co-infected children against without infection or with single *H. pylori* infection.

Groups	No./total	PR (95% CI)	p
*H. pylori* and EBV MN, severe			
*HP*+/EBV+ *vs HP*−/EBV−	21/125 *vs* 0/66	Undefined	0.0004
*HP*+/EBV+ *vs HP*+/EBV−	21/125 *vs* 4/53	2.23 (0.7–5.8)	0.15
PMN, moderate-severe			
*HP*+/EBV+ *vs HP*−/EBV−	27/125 *vs* 0/66	Undefined	0.00004
*HP*+/EBV+ *vs HP*+/EBV−	27/125 *vs* 3/53	4.1 (1.2–23.5)	0.01
CagA and EBV MN, severe			
CagA+/EBV+ *vs HP* CagA−/EBV−	18/78 *vs* 1/19	5.4 (0.7–43.3)^a^	0.08
CagA+/EBV+ *vs HP* CagA+/EBV−	18/78 *vs* 3/34	3.0 (0.8-11.1)	0.08
PMN, moderate-severe			
CagA+/EBV+ *vs HP* CagA−/EBV−	25/78 *vs* 1/19	8.5 (1.1–67.2)^a^	0.01
CagA+/EBV+ *vs HP* CagA+/EBV−	25/78 *vs* 2/34	7.2 (1.6–31.7)	0.003

a This estimation could not be adjusted by age because there are 0 cases of severe MN or moderate-severe PMN infiltration in the ≤10 years of age group.

When cases with co-infection (CagA+/EBV+) were compared against cases of only *H. pylori* infection (*HP* CagA−/EBV−) PR's were 5.4 for MN and 8.5 for PMN. Similarly, when patients with co-infection (CagA+/EBV+) were compared against cases with single *H. pylori* CagA+ infection (CagA+/EBV−PR's were 3.0 for MN and 7.2 for PMN (***Table*** **** ***4***). The level of PMN infiltration also showed statistical significance supporting that co-infection is necessary to develop severe gastritis in children, and thus pointing out for a critical role for EBV that cannot be provided even by the CagA virulence factor (p = 0.003).

## Discussion

*H. pylori* infection is acquired early in life during childhood, and the presence of the bacteria induces an inflammatory reaction in the gastric mucosa which in most cases causes no disease. However, in some individuals the chronic long lasting inflammation triggers serious damage to the gastric epithelium increasing the risk to develop precancerous lesions, which in turn increase the risk to end up with a life threatening GC.

Some of the co-factors that promote a more severe inflammatory reaction may present early on during childhood. The inflammatory nature of GC denotes a critical role for an immunogenic agent and in accordance with this, GC is a cancer primarily of infectious etiology. While the association of *H. pylori* with GC and early inflammatory lesions is well documented, only a few studies have analyzed the participation of EBV infection in patients with gastritis, three of them in adults over 40****years old [22], [23], [24] and two case reports in young women of 18 [25] and 17****years old [26]. To our knowledge, the latter is the only case of EBV and gastritis reported to date in a pediatric patient in which high serum levels of anti-VCA antibodies (IgG and IgM) were found. Other studies have examined the presence of EBV sequences in gastritis samples. A recent report found EBV sequences by quantitative PCR in 15/50 and 5/6 of pediatric and adult gastritis, respectively [27]. Another recent study in adults found that about 90% (12/13) of chronic atrophic gastritis present EBV sequences [28]. Taken together these data support an important role for EBV in early inflammatory reactions of the gastric mucosa.

Studies in adults with NPC have found that disease progression correlates with increased antibodies against EBV reactivation antigens [10], [16], [17]. We hypothesized that a similar phenomenon may occur in GC and that the study of children with EBV infection may help identify patients with severe inflammation in the gastric mucosa, potentially at higher risk to develop precancerous lesions. Antibodies against proteins of the EBV lytic cycle could reflect higher levels of infection of the upper digestive tract epithelia and thus serve as a marker for the lesion progression. However, when we analyzed EBV infection in children, we did not observe the expected correlation with severe gastritis, and higher levels of anti-EBV antibodies either IgG or IgM, reflecting acute or chronic infections, were found preferentially in cases with mild gastritis. Thus, EBV single infection does not explain the cases of severe gastritis.

Until now, studies have been limited to *H. pylori* infection and have shown that in children it is usually associated with a mild to moderate inflammation (MN cell infiltration) and a mild to absent activity (PMN cell infiltration), although a severe inflammation and activity is observed in a few cases. When we analyzed co-infection with both *H. pylori* and EBV we found that children infected with both pathogens had the strongest association with severe gastritis, as measured by severe infiltration of MN (inflammation) and PMN cells (activity) in the gastric mucosa. In contrast, patients *HP*−/EBV+ preferentially presented mild inflammation (MN cell infiltration) and no activity (absence of PMN cells); while those *HP*+/EBV− presented moderate inflammation and mild activity. Our results suggest that co-infection with EBV and *H. pyori* is necessary to cause severe gastritis, supporting an important role for EBV, at least in pediatric patients. This increased effect was also true for patients infected with *H. pylori* CagA+ strains, already known to be associated with greater virulence and increased risk for GC. In our analyses, children co-infected with CagA+/EBV+ showed a significantly stronger association with severe gastritis than infected with CagA+/EBV−, further supporting the need of EBV to cause severe gastritis even in the presence of highly pathogenic *H. pylori* CagA+ strains. Interestingly, co-infection by EBV and *H. pylori* CagA negative strains did not trigger severe stages of gastritis, arguing against EBV complementing the pathogenesis of less virulent CagA negative *H. pylori* strains and thus still supporting the importance of CagA.

It is postulated that in order to infect epithelial cells, EBV must exit first from B cell latency, a step triggered by expression of transcriptional factor Zta, which is the main orchestrator of the lytic cycle. However, the extracellular signals in the upper digestive tract that trigger Zta expression are unknown. CagA is an oncoprotein that among many documented functions induces loss of polarity in epithelial cells, allowing the transport of basolateral proteins towards the apical face [29]. It might be possible that CagA signaling could function as trigger of the EBV lytic cycle, and CagA induced loss of cell polarity could also favor EBV tropism for epithelial cells. Arguing against the latter, we observed more pediatric patients infected with EBV (214 cases) than with *H. pylori* (178 cases), which might indicate that infection with the bacteria does not necessarily occur first.

It is important to address how EBV and *H. pylori* interact in the gastric mucosa in future studies. Two possible mechanisms are envisioned: one is simply through additive inflammatory responses causing increased damage to the tissue. A second mechanism is through more intimate interactions between EBV and *H. pylori* genes, *e.g.* the above-mentioned putative CagA and Zta cooperation. *H. pylori* infection has also been positively linked to MALT gastric lymphoma [30] supporting increased activation/signaling of B cells transiting through the gastric mucosa. In this scenario, one of the main inducers of Zta expression and the EBV lytic cycle in B cell cultures is protein kinase C (PKC) [31], [32], and CagA is a known activator of this kinase [33]. Ectopic expression of CagA in transgenic mice supports that this protein is the first described bacterial oncoprotein and as such it behaves similarly to viral oncoproteins [8], [9]. Interestingly, CagA and EBV oncogenes LMP1 and LMP2A trigger activation of NFκB and MAP Kinases, important signaling pathways for increased cell survival and proliferation during oncogenic transformation [34], [35]. The common activation of signaling pathways by both pathogens suggests a common mechanism of infection/transformation of the gastric epithelium.

Although our data supports that co-infection of EBV with *H. pylori* is necessary to cause severe gastric inflammation and activity, EBV requirement seems to be greater for induction of severe activity in the gastric mucosa. Severe activity is given by a large infiltrate of polymorphonuclear cells permeating the lamina propria and the gastric glands, which in turn activate and secrete more pro-inflammatory mediators, thus enhancing and perpetuating the local inflammatory reaction and increasing the risk for severe and permanent damage to the gastric mucosa. In *H. pylori* infection, IL-8 seems to be the main neutrophil attracting cytokine [4], [36], [37]. IL-1β has been found overexpressed in NPC tumor samples, an important cytokine for PMN recruiting [38]. It is possible that the combined role of IL-8 and IL-1β results in the increased activity observed in EBV and *H. pylori* co-infected patients.

In conclusion, our results strongly suggest that EBV is involved in the pathophysiology of severe gastric lesions in children infected with *H. pylori*. Our results also suggest that in the absence of EBV infection, *H. pylori* is not sufficient to trigger severe gastritis in children. This study points out the need to study both pathogens jointly to understand the mechanism behind severe damage of the gastric mucosa early in life, which could have implications for the correct disease diagnosis and treatment; also, it could help to identify children with increased risk to present more serious lesions later in life.

## Supporting Information

Table S1 Cumulative percentages of cases and *H. pylori* and EBV seroprevalence.
(DOCX)

Table S2 Correlation between antibody titers and the gastritis severity.
(DOCX)
