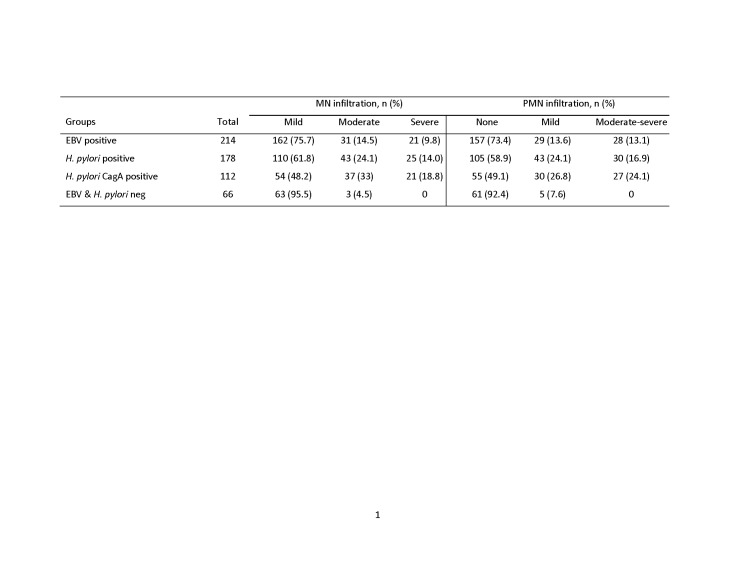# Correction: Epstein Barr Virus and *Helicobacter pylori* Co-Infection Are Positively Associated with Severe Gastritis in Pediatric Patients

**DOI:** 10.1371/annotation/865eaad7-8547-49ac-a42d-47e9d0755bb3

**Published:** 2013-11-08

**Authors:** María G. Cárdenas-Mondragón, Ricardo Carreón-Talavera, Margarita Camorlinga-Ponce, Alejandro Gomez-Delgado, Javier Torres, Ezequiel M. Fuentes-Pananá

Errors were introduced in the preparation of this article for publication. The last line of values in Table 2, group of EBV & H. pylori neg, is not complete. The complete Table 2 can be viewed here: 

**Figure:**