# Supplementary material for: Epstein Barr Virus and Helicobacter pylori Co-Infection Are Positively Associated with Severe Gastritis in Pediatric Patients
Source: PLoS One. 2013 Apr 24;8(4):e62850. doi: 10.1371/journal.pone.0062850 (PMC3634751; doi:10.1371/journal.pone.0062850)
Supplement: Table S1 — Cumulative percentages of cases and H. pylori and EBV seroprevalence. (DOCX) [file pone.0062850.s001.docx]

**Table S1**. Cumulative percentages of cases and *H. pylori* and EBV seroprevalence.

| Age | | | No. cases | | Cumulative percentage | No. cases  *Hp+* | | Cumulative percentage  *Hp+* | | No. cases EBV+ | Cumulative percentage EBV+ | |  |
| --- | --- | --- | --- | --- | --- | --- | --- | --- | --- | --- | --- | --- | --- |
|  | 0 | 2 | | .6 | | 0 | 0 | | 1 | | | 0.5 | |
|  | 2 | 5 | | 2.1 | | 3 | 1.7 | | 3 | | | 1.9 | |
|  | 3 | 7 | | 4.2 | | 4 | 3.9 | | 2 | | | 2.8 | |
|  | 4 | 10 | | 7.2 | | 4 | 6.2 | | 5 | | | 5.1 | |
|  | 5 | 22 | | 13.8 | | 12 | 12.9 | | 16 | | | 12.6 | |
|  | 6 | 20 | | 19.8 | | 6 | 16.3 | | 10 | | | 17.3 | |
|  | 7 | 28 | | 28.2 | | 14 | 24.2 | | 12 | | | 22.9 | |
|  | 8 | 17 | | 33.3 | | 9 | 29.2 | | 13 | | | 29 | |
|  | 9 | 27 | | 41.4 | | 16 | 38.2 | | 16 | | | 36.4 | |
|  | 10 | 29 | | 50.2 | | 18 | 48.3 | | 22 | | | 46.7 | |
|  | 11 | 36 | | 61.0 | | 17 | 57.9 | | 21 | | | 56.5 | |
|  | 12 | 29 | | 69.7 | | 11 | 64 | | 23 | | | 67.3 | |
|  | 13 | 26 | | 77.5 | | 15 | 72.5 | | 20 | | | 76.6 | |
|  | 14 | 33 | | 87.4 | | 17 | 82 | | 21 | | | 86.4 | |
|  | 15 | 18 | | 92.8 | | 13 | 89.3 | | 11 | | | 91.6 | |
|  | 16 | 20 | | 98.8 | | 17 | 94.9 | | 15 | | | 95.8 | |
|  | 17 | 4 | | 100.0 | | 2 | 100 | | 3 | | | 100 | |
|  | Total | 333 | |  | | 178 |  | | 214 | | |  | |
