# Supplementary material for: Epstein Barr Virus and Helicobacter pylori Co-Infection Are Positively Associated with Severe Gastritis in Pediatric Patients
Source: PLoS One. 2013 Apr 24;8(4):e62850. doi: 10.1371/journal.pone.0062850 (PMC3634751; doi:10.1371/journal.pone.0062850)
Supplement: Table S2 — Correlation between antibody titers and the gastritis severity. (DOCX) [file pone.0062850.s002.docx]

**Table S2.** Correlation between antibody titers and the gastritis severity.

| **Variable** | **Total** | **MN cell infiltration** | | |  | **PMN cell infiltration** | | |
| --- | --- | --- | --- | --- | --- | --- | --- | --- |
|  |  | **Mild** | **Moderate** | **Severe** |  | **None** | **Mild** | **Moderate-severe** |
| **Group** | median (range) | median (range) | median (range) | median (range) |  | median (range) | median (range) | median (range) |
| **EBV & *HP* pos**  EBV IgG | 61.6 (21.3-148.3) | 59.7 (21.3-140.6) | 63.5 (21.3-148.3) | 55.1 (23.1-137.3) |  | 59.2 (21.3-144.9) | **73.5 (25.4-148.3)** | 53.7 (21.3-116.5) |
| **EBV pos & *HP* neg**  EBV IgG | 62.1 (21.3-159.7) | 60.6 (21.3-159.7) | 84.7 (71.6-97.9) | 0 |  | 61.7 (21.3-159.7) | 61.5 (24.9-88.8) | 97.9 |
| **EBV pos & *HP* CagA pos**  EBV IgG | 60.6 (22.5-148.3) | 58.7 (22.5-140.6) | 62.1 (21.3-148.3) | 53.7 (30.3-116.53) |  | 58.7 (22.5-144.9) | **83.1 (25.4-148.3)** | 53.7 (21.3-116.5) |
| **EBV pos & *HP*** **CagA neg**  EBV IgG | 67.1 (21.3-140.5) | 63.2 (21.3-140.5) | 79.5 (35.9-111.3) | 95.8 (23.1-137.3) |  | 61.8 (21.3-140.5) | 70.6 (26.9-137.3) | 59.4 (23.1-95.8) |
| ***H. pylori* pos**  ***HP*** IgG | 1.8 (1-11.8) | 1.4 (1-11.8) | 2.2 (1-8.3) | **2.4 (1-9.7)** |  | 1.5 (1-10.8) | 1.9 (1-11.8) | **2.4 (1-9.7)** |

Numbers in bold denote statistical significance (p ≤ 0.05)
